# Multilevel L4 and L5 Corpectomy for Burst Fracture via an Anterior Transperitoneal Approach Followed by Posterior Stabilization: Technical and Anatomic Considerations

**DOI:** 10.7759/cureus.18579

**Published:** 2021-10-07

**Authors:** Kelly Gassie, Young Erben, Susana Fortich, Gian P Carames, Sukhwinder Johnny S Sandhu, Kingsley Abode-Iyamah

**Affiliations:** 1 Neurosurgery, Mayo Clinic, Jacksonville, USA; 2 Vascular and Endovascular Surgery, Mayo Clinic, Jacksonville, USA; 3 Radiology, Mayo Clinic, Jacksonville, USA

**Keywords:** anterior approach, abdominal, lumbar vertebra, anterior transperitoneal exposure, corpectomy

## Abstract

Lower lumbar spine burst fractures make up only 1% of all lumbar spine fractures. A burst fracture with neurological compromise, ligamentous injury, severe kyphotic deformity, or loss of anterior column support typically requires surgical stabilization. Treatment options at the L4 and L5 levels are challenging and often require an anterior/posterior approach. Very little has been reported on anterior approaches to the L4 and L5 levels when a corpectomy is required. Hence, we present a patient with a complex burst fracture of L4 and L5. She underwent a corpectomy of L4 and L5 and placement of an expandable cage through a window created between the aorta and the inferior vena cava via an anterior transperitoneal abdominal approach followed by posterior stabilization and fusion from L2 to the pelvis.

## Introduction

Lower lumbar spine burst fractures (L4 and L5) make up only about 1% of all lumbar spine fractures [1,2]. Typically, these fractures result from high energy trauma, including motor vehicle accidents or falls, but can also be seen in patients with osteoporosis [2,3]. The biomechanical properties of the lower lumbar spine make treatment of burst fractures in this area more challenging. Particularly, the L4 and L5 lumbar vertebral bodies play a major role in the axial weight-bearing properties of the spine and are responsible for maintaining lumbar lordosis [2,4]. Thus, burst fractures at these levels can lead to loss of lordosis, additionally altering the biomechanical properties of the spine [4].

A burst fracture with neurological compromise, ligamentous injury, severe kyphotic deformity, or loss of anterior column support typically requires surgical stabilization. Fractures that require operative intervention include those with 40% or more canal compromise, 25 degrees or more kyphosis, and more than 50% loss of vertebral height [5,6]. Treatment options at the L4 and L5 levels are challenging when this occurs and often require an anterior/posterior approach [7]. Very little has been reported on anterior approaches to the L4 and L5 levels when a corpectomy is required. This is because access to these levels can be difficult due to the positioning of the aorta and inferior vena cava [2].

Herein, we report a patient with a severe burst fracture of the L4 vertebral body with associated severe L4-5 spinal canal stenosis that was surgically managed with a corpectomy of L4 and L5 through an anterior abdominal exposure followed by posterior stabilization. The patient had significant loss of anterior column support, severe canal compromise, ligamentous injury, and neurologic deficits. A posterior-only approach for stabilization was not feasible because of her prior L3-L5 laminectomies. We believe this is one of the first reports of a multilevel lumbar spine corpectomy for a burst fracture through an anterior approach traversing the dangerous corridor of vessels including the inferior vena cava, iliac veins, aorta, and the aortic bifurcation.

## Case presentation

**Figure 1:**
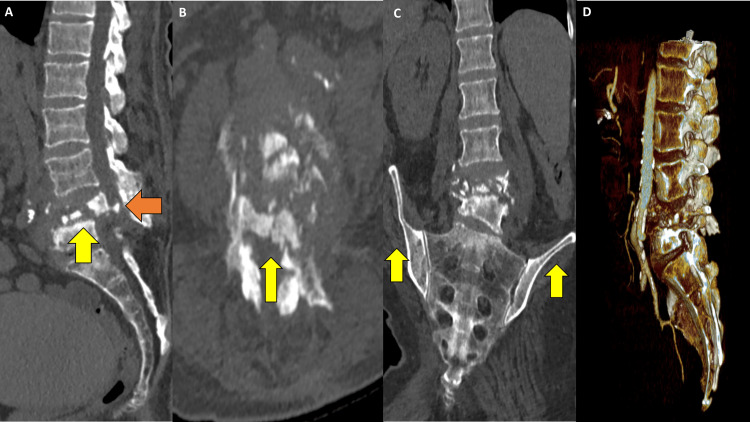
A) Sagittal multiplanar reconstruction of lumbar CT demonstrating a two-column L4 burst fracture with retropulsed fracture fragments traverse the complete anterior-posterior (AP) spinal canal (orange arrow). There are changes of bone resorption with two-column fracture of also the L5 superior endplate (yellow arrow); B) Axial view at the L4-5 level showing severe canal compromise (yellow arrow); C) Coronal view showing the right pelvic and lumbar tilt (yellow arrows) due to the burst fracture; D) Volume-rendered 3D reconstruction with sagittal clip plane through the left lateral vertebral body plane.

**Figure 2:**
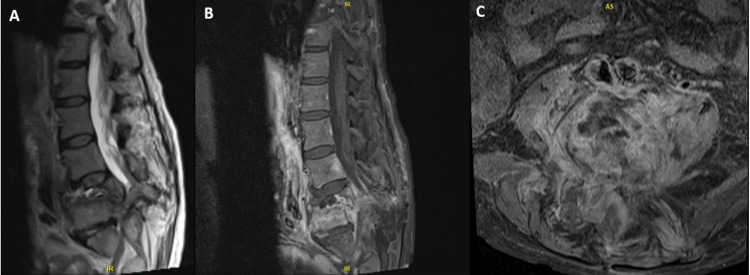
A) Sagittal T2 Turbo Spine Echo (TSE) MRI of the lumbar spine demonstrates the L4 and L5 fractures with near complete loss of the intrathecal subarachnoid space. B and C) Sagittal and Axial T1 TSE DIXON water images with fat suppression were obtained of the lumbar spine after contrast administration. Images demonstrate associated enhancement consistent with inflammatory changes but no epidural abscess or focal mass lesion.

A 59-year-old female patient with a BMI of 26.9 kg/m2 and a significant past medical history of lung cancer and a motor vehicle accident over 10 years prior presented to the emergency department due to acute chronic back pain with worsening numbness and weakness in her lower extremities. She had multiple back operations including an L3-L5 laminectomy performed two months at another institution prior to presentation without resolution of symptoms. On examination, her lower extremities were intact except for bilateral 3/5 strength in her anterior tibialis and extensor hallucis longus. An initial computed tomography (CT) scan (Figure 1) revealed a two-column burst fracture at L4 with 9mm retropulsed fracture fragments that resulted in severe, nearly complete, spinal canal stenosis. Two-column fracture also involved the L5 level with extension into the left L5 pedicle. There was associated significant bony resorption at L4 and L5. A magnetic resonance imaging (MRI) of the spine with and without contrast (Figure 2) was obtained to further assess the ligamentous structures, spinal canal, and nerve roots. MRI was also obtained to evaluate for possible associated neoplastic or infectious etiologies. A multi-disciplinary approach to care, including vascular surgery, neuroradiology, and neurosurgery recommended a corpectomy with subsequent posterior stabilization. The corpectomy was performed through an anterior approach with a staged posterior L2-pelvis instrumentation five days later.

**Figure 3:**
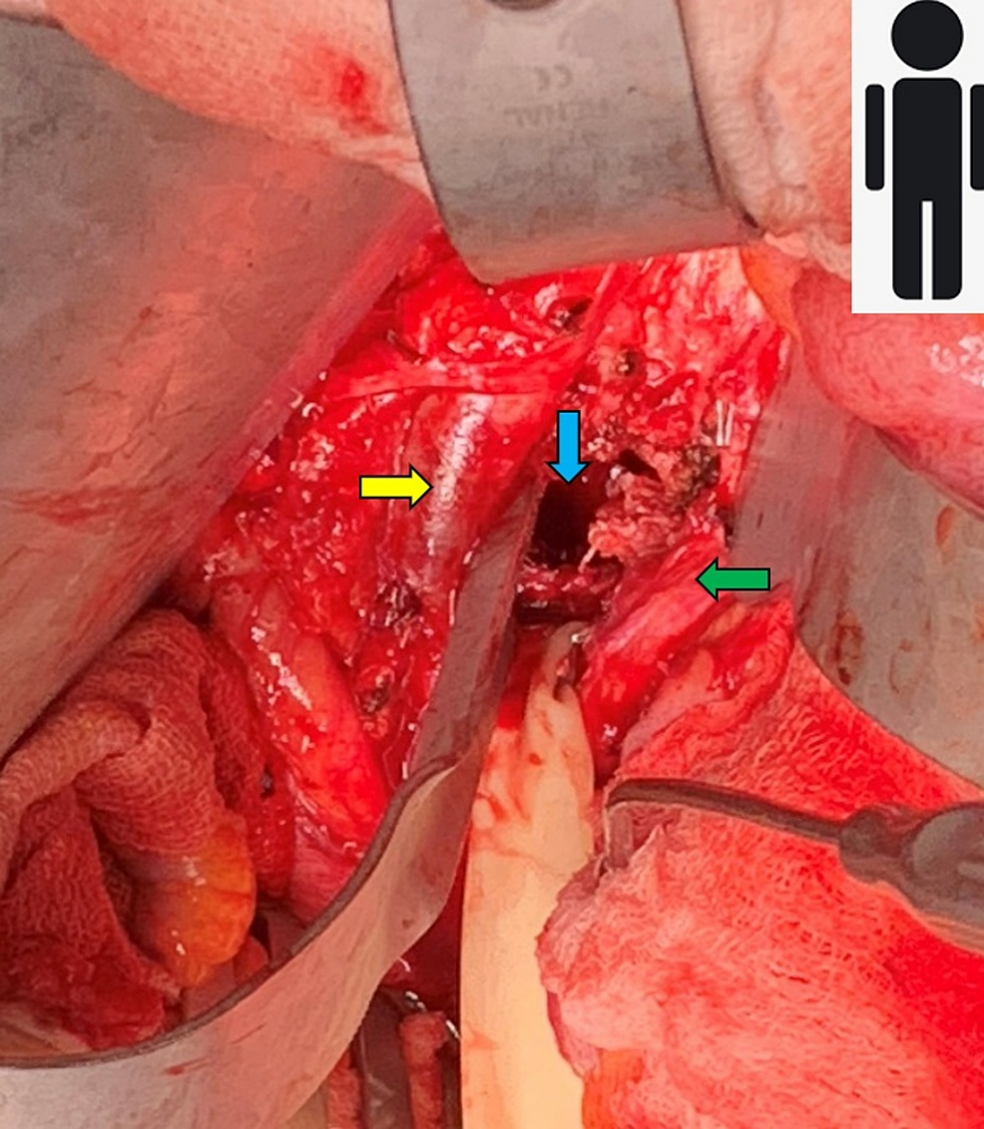
Intra-operative view of corpectomy of L4 and L5 (blue arrow). Inferior vena cava (yellow arrow) and aorta (green arrow) both retracted laterally.

**Figure 4:**
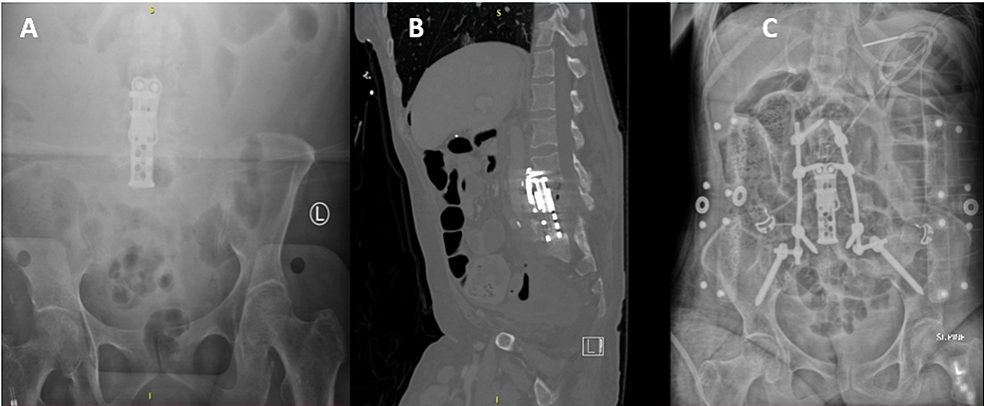
A) Post-operative abdominal supine radiograph in the AP projection  and B) Post-operative sagittal multiplanar reconstruction of the lumbar spine from an abdominal pelvic computed tomography demonstrating anterior stabilization of the spine using a K2M Capri Corpectomy cage and C) Post-operative abdominal supine AP radiograph demonstrating anterior and posterior stabilization

A standard abdominal aortic exposure was performed by the vascular surgery team. Once the aorta, inferior vena cava, lumbar arteries, and veins were exposed; most of these small branches were ligated in continuity; which allowed for further easier exposure of the spine by carefully retracting both the aorta and inferior vena cava laterally in opposite directions. Through a small window between the aorta and the inferior vena cava, we were able to expose the anterior spine, which was significantly scarred and inflamed. We then proceeded with the identification of the L3-L4 disk space under fluoroscopy. Working between the aorta and the inferior vena cava, the L3-4 and L5-S1 discs were exonerated. Using small curettes and an M8 drill bit, we then performed the L4 and L5 corpectomy (Figure 3). The retropulsed fracture fragments of L4-5 were meticulously dissected away from the dura until the thecal sac was decompressed. Once the endplates above and below our levels were prepared, we carefully placed a Stryker Capri Corpectomy cage (17x22mm) filled with Vivigen (DePuy Sytheses, Warsaw, IN, USA), with the capability to expand from 58-72mm and a variable degree between the L3 and S1 endplates. We placed the cage from cranial to caudal passing the cage underneath the vessels. Using intraoperative fluoroscopy, we then expanded the cage to 64mm. Subsequently, a 47-mm anterior plate was placed and secured with 20-mm screws at the L3 level (Figure 4A-4B). The vascular team then proceeded to close the retroperitoneal space using a bovine pericardium sheath (Edwards Lifesciences Corp., Irvine, CA, USA) to prevent possible future fistula formation. The operation duration was 10 hours and 41 minutes with an estimated blood loss of 1300 ml. Five days later, the patient was taken back for a posterior fusion from L2 to the pelvis (Figure 4C). The patient tolerated both operations well. She was treated for multidrug-resistant Klebsiella pneumonia with vancomycin and ertapenem for six weeks. After 14 days, she was deemed stable for discharge to a rehabilitation facility. The patient had a two-month follow-up visit and she was progressing well recovering from her operations.

## Discussion

Burst fractures are defined as involving the anterior and middle columns of the spine using the Denis model [8]. The mechanism is typically axial loading with undue flexion causing fractures of the vertebral body involving both columns. However, there are many classification systems used. The Arbeitsgemeinschaft für Osteosynthesefragen (AO) Spine classification system attempts to classify fractures and guide treatment by simplifying criteria. Type A injuries indicate compression, type B injuries include distraction and type C injuries define translational fractures. An A3 burst fracture involves only one endplate, while an A4 burst fracture involves both endplates [9]. Typically burst fractures are similar to compression fractures, however, burst fractures involve the posterior wall of the vertebral body with retropulsed bone to varying degrees, sometimes causing neurological injury [1]. Burst fractures have no ligamentous injury involvement, and if so, would transition the categorization of the injury to flexion-distraction, type B, and likely surgical stabilization.

Treatment of burst fractures is an area of debate amongst spine surgeons. The Thoracolumbar Injury Classification System (TLICS) is frequently used to direct treatment involving spine fractures. The TLICS bases criteria amongst three parameters including the morphology of the fracture, neurological injury/status of the patient, and integrity of the posterior-ligamentous capsule [10]. Scores are given to each of these criteria, in which a score of 5 or more indicates surgical treatment. There have been many studies that have shown good outcomes with conservative treatment in burst fractures in which there are no neurological deficits, ligamentous injury, severe pain, or kyphosis at the fracture level [5,10-19]. However, burst fractures of the L4 and L5 levels are rarer, and studies are limited in terms of management and outcomes. Even more uncommon are multilevel anterior lumbar corpectomies.

The surgical approach for pathologies of the L5 vertebra is recognized for being one of the most challenging surgical scenarios for any spine surgeon [15,20]. The anatomical features of the lumbosacral junction employ high-level stress on surgical constructs; sliding and compressive forces are key factors that contribute to this challenging procedure [15]. In a study by Blanco et al. from 2005, authors published a retrospective report of five cases of isolated L5 burst fractures without neurological deficit all managed with protected mobilization. The authors concluded that in L5 burst fractures without canal compromise, little deformity, and no neurologic compromise, conservative management was appropriate [21]. There have been few articles describing surgical management in lower lumbar (L4 and L5) burst fractures. Mootaz et al. reported clinical and radiographic outcomes with L5 corpectomies in 25 cases. Twenty-four of the 25 patients had anterior approaches to cage placement, in which the authors describe the demanding procedure and expectation of large amounts of intraoperative blood loss [19]. There are many other reports of single-level corpectomies of the L4 and L5 vertebral bodies [22-27], however, there are no anatomical reports of anterior approaches for multilevel lower lumbar spine corpectomies.

The vascular anatomy of the lumbosacral region with the left common iliac vein crossing the spine diagonally anterior to the L5 vertebra makes the anterior exposure one of the most difficult approaches to perform. Likewise, intraoperative vascular injuries are the most frequently encountered lesions during anterior spine procedures with a prevalence that varies from 7.9% to 13.8% [19]. Additionally, lumbosacral spine access from an anterior approach can be obtained via several incisions [24]. Many surgeons prefer a right paramedian incision close to the midline to avoid injury to the nerves innervating the rectus, an oblique incision running from the iliac crest to a point between the umbilicus and pubis, or a transverse incision with either a muscle-cutting or muscle-splitting approach. When a patient has several previous retroperitoneal surgeries, like our patient, the transperitoneal approach can be used. Once the surgeon has access to the peritoneal cavity, the retroperitoneal space is accessed by mobilizing the small bowel laterally, and once in this space, the exposure of the lumbar discs is accessible [24].

Lately, a new technique involving minimally invasive surgery has been described. Le et al. reported a retrospective case series with good results. They performed 20 cases of minimally invasive thoracolumbar corpectomy in a period of four years. They included 12 men (60%) and eight women (40%) with a mean age of 54.3 years. Indications for surgery were infection (n=6, 30%), metastatic disease (n=2, 10%), fracture (n=6, 30%), and calcified disc herniation (n=6, 30%). Partial and complete corpectomy was performed in five patients (25%) and 15 patients (75%), respectively. Estimated blood loss was 558.4 mL. Mean length of stay from admission and surgery was 14.6 and 11.4 days, respectively. Mean length of stay from surgery for elective cases was 4.2 days. Visual analogue scale score in terms of pain improved from 7.7 to 4.5 (P<0.01). There was a total of three postoperative complications in two patients, including one mortality for urosepsis. One patient had revision spinal surgery for adjacent segment disease [28]. Because our patient previously had several spine procedures, a transperitoneal anterior approach was felt to be the most appropriate technique to address the corpectomy of L4 and L5. To the best of our knowledge, there is no previous case reported about this approach and more studies are required to determine the safety and effectiveness of this unique technique.

## Conclusions

Lower lumbar spine burst fractures with neurological sequela, loss of anterior column support, and ligamentous stability should be treated surgically. When a corpectomy is required, an anterior approach to the L4 and L5 vertebral bodies is feasible. However, working between the narrow vascular corridors makes this approach high risk. Understanding the anatomical considerations of a transperitoneal approach is key when performing a multilevel corpectomy at these levels. 
